# PCR-detectable *Candida* DNA exists a short period in the blood of systemic candidiasis murine model

**DOI:** 10.1515/biol-2020-0075

**Published:** 2020-09-06

**Authors:** Zheng-Xin He, Hui-Hai Zhao, Fu-Kun Wang

**Affiliations:** Department of Clinical Laboratory, The 980th Hospital of PLA Joint Logistical Support Force (Bethune International Peace Hospital), 398 Zhongshan Road, Shijiazhuang, Hebei, 050082, People's Republic of China

**Keywords:** *Candida albicans*, qPCR, candidiasis murine model, DNA preparation, invasive candidiasis

## Abstract

Invasive candidiasis is a major challenge to clinical medicine today. However, traditional fungal diagnostic techniques and empirical treatments have shown great limitations. Although efforts are necessarily needed in methodology standardization and multicenter validation, polymerase chain reaction (PCR) is a very promising assay in detecting fungal pathogens. Using a “heat-shock” DNA preparation method, a rapid and simple PCR protocol for quantification of the *Candida albicans* (*C. albicans*) ribosomal DNA was established. The PCR assay could detect *Candida* DNA as low as 10 CFU/mL in samples prepared by the heat-shock protocol, without any cross-reaction with DNA prepared from other *Candida* spp. and bacterial pathogens. For simulated blood samples, the PCR test sensitivity of whole blood samples was better than that of plasma and blood cells. In the systemic candidiasis murine model, detectable DNA was only observed within 24 h after *C. albicans* SC5314 injection, which is much shorter than that observed in the kidney.

## 1 Introduction

Clinical medicine is facing increasing challenges of invasive candidiasis (IC). *Candida* spp. is the main pathogen of serious fungal infections, causing about 10–15% of hospital-acquired urinary tract fungal infections [1]. Currently, candidemia is the fourth leading cause of hospital-acquired bloodstream infections [2], and about 1 million people worldwide die from IC every year. However, numerous studies still speculate that this figure is seriously underestimated [3]. Candidiasis is also associated with increased healthcare costs, and the attributable cost of candidiasis is reported to be more than US$40,000 per patient [4]. IC is usually a consequence of increased or abnormal colonization together with defect in host defenses. Common risk factors include indwelling central venous catheter, broad-spectrum antibacterial agents, long-term intensive care unit stay, total parenteral nutrition, iatrogenic immunosuppression, etc. [5]. It should be noted that fungal infections caused by new *Candida* spp. have risen rapidly, and some have even appeared as large-scale outbreaks and regional epidemics, which need urgent attention. For example, the “superbug” *Candida auris*, a multidrug-resistant fungus, has recently caused serious outbreaks in USA [6]. Until March 2019, 613 confirmed cases and 1,123 colonized patients were reported in the United States, most of which are critical patients [7,8].

Traditional fungal diagnostic techniques and empirical treatments have shown great limitations when facing the IC challenge. Fungi cultivation is the gold standard for the diagnosis of IC, while this method has major drawbacks such as being time- and labor-consuming. Over the past few decades, diverse non-culture laboratory methods have been developed for rapid IC diagnosis [9,10]. Nowadays, the (1,3)-β-d-glucan (BDG) test kits have been approved and commercially available in several countries including China. The European Society of Clinical Microbiology and Infectious Diseases guidelines recommend using the BDG test in diagnosing IC [11]. However, the BDG is not a species-specific test and cannot discriminate infections caused by different fungal pathogens, resulting in difficulties in designing specific management protocol in clinic. Furthermore, false-positive results due to contamination are not rare in using BDG assays [12,13].

Polymerase chain reaction (PCR) is a promising assay in detecting fungal pathogens with the advantages of high throughput, high sensitivity and specificity. A meta-analysis which included 54 different studies showed a pooled sensitivity and specificity of 0.95 and 0.92, respectively, for the diagnosis of candidemia [14]. Still, PCR assays have drawbacks such as the requirement of sophisticated equipment, labor-intensive and prolonged time to results. In the present study, we detected *Candida albicans* (*C. albicans*) in a systemic candidiasis murine model by the TaqMan PCR assay. We have attempted to find an easy protocol to determine *C. albicans* in the blood samples.

## 2 Materials and methods

### 2.1 *Candida* strain and culture condition

*C. albicans* strain SC5314, a human clinical isolate recovered from a patient with generalized candidiasis [15], has been preserved in our laboratory [16]. In this study, *C. albicans* SC5314 was cultured in yeast extract peptone dextrose medium for 48 h at 37°C. Fungal cells were serially diluted (tenfold) from 1 × 10^5^ to 1 CFU/mL with sterile saline suspensions after counting with a hemocyte counter.

### 2.2 Murine model of systemic candidiasis

The systemic candidiasis murine model was developed as described in our previous work [16]. Briefly, mice were injected with cyclophosphamide intraperitoneally (i.p.) (Jiangsu Hengrui Medicine Co. Ltd., China) at a dose of 100 mg kg^−1^ before *C. albicans* challenge. Four days later, the mice received 1 × 10^6^ CFU of *C. albicans* SC5314 (in 0.1 mL normal saline [NS]) injection i.p., and 0.1 mL of sterile NS was injected as a negative control. Three mice were randomly chosen and sacrificed at the time point of 1 h, 6 h, 12 h, 1 d, 2 d and 5 d after *C. albicans* challenge. The mice blood samples were collected in EDTA-K2 tubes.


**Ethical approval:** The research related to animal use has been complied with all the relevant national regulations and institutional policies for the care and use of animals and has been approved by the Bethune International Peace Hospital Animal Care and Use Committee.

### 2.3 Seeded human blood samples

*C. albicans* cells were added to 1 mL of anticoagulated blood to final concentrations from 1 × 10^4^ to 10 CFU/mL to mimic clinically infected blood samples. The blood samples were incubated in 37°C for 24 h. After the incubation, serum was separated by centrifugation and the isolated blood cells were lysed using a Cytolysis Kit (Sansure Biotech, Changsha, China). Unlysed sediments were collected, washed and resuspended in 400 µL of nucleic acid-releasing reagent (Sansure Biotech, Changsha, China).


**Informed consent:** Informed consent has been obtained from all individuals included in this study.
**Ethical approval:** The research related to human use has been complied with all the relevant national regulations, institutional policies and in accordance with the tenets of the Helsinki Declaration and has been approved by the Ethics Committee of Bethune International Peace Hospital.

### 2.4 Heat-shock

The *C. albicans* suspensions or unlysed blood sediments were heated at 100°C for 15 min and then transferred to −80°C for 10 min (repeated three times). DNA of *C. albicans* was extracted using the TIANamp Yeast DNA Kit (Tiangen Biotech, Beijing, China). The extracting procedure was performed according to the manufacturer’s recommendations. Briefly, solutions of 200 μL of GA buffer, 20 μL of proteinase K and 220 μL of GB buffer were added to the collected yeast cells and incubated at 70°C for 10 min, and mixed well to lyse the cells adequately. Then, the yeast DNA was precipitated with 220 μL of ethanol and adsorbed with DNA-adsorbing column. After rinsing twice with 70% ethanol solution, elution was performed with 50 µL of Tris-EDTA buffer.

### 2.5 Quantitative PCR (qPCR)

qPCR assay was performed to detect *C. albicans*. Primers and probe were designed on the basis of the nucleotide sequences of the ITS2 ribosomal DNA region from the SC5314 strain (NCBI Reference Sequence: NC_032096.1). Forward: 5′-GGT GTT GAG CAA TAC GAC-3′; reverse: 5′-AGA CCT AAG CCA TTG TCA-3′; TaqMan probe: 5′-FAM-ATC CCG CCT TAC CAC TAC CG-TAMRA-3′. The primers and probe were synthesized by Sangon Biotech (Shanghai, China).

DNA samples were analyzed using the SLAN-96P Real-Time PCR System (Hongshi Medical Technology Co., Ltd., Shanghai, China). Each 50 µL of the TaqMan PCR mixture contained 5 µL aliquot of sample DNA, 25 µL of 2× Quant One Step qRT-PCR Master Mix (Tiangen Biotech, Beijing, China), 2 µL of Hot Taq polymerase (2.5 U µL^−1^), 2 µL of each primer (0.4 µM), 1 µL of TaqMan probe and 13 µL of deionized-distilled water. The cycling conditions were set as follows: an initial denaturation at 95°C for 2 min, 40 cycles of denaturation at 95°C for 15 s and annealing at 60°C for 1 min. Threshold (*C*_t_) values were calculated by the PCR system automatically.

## 3 Results

### 3.1 Sensitivity and specificity of the TaqMan qPCR assay

**Figure 1:**
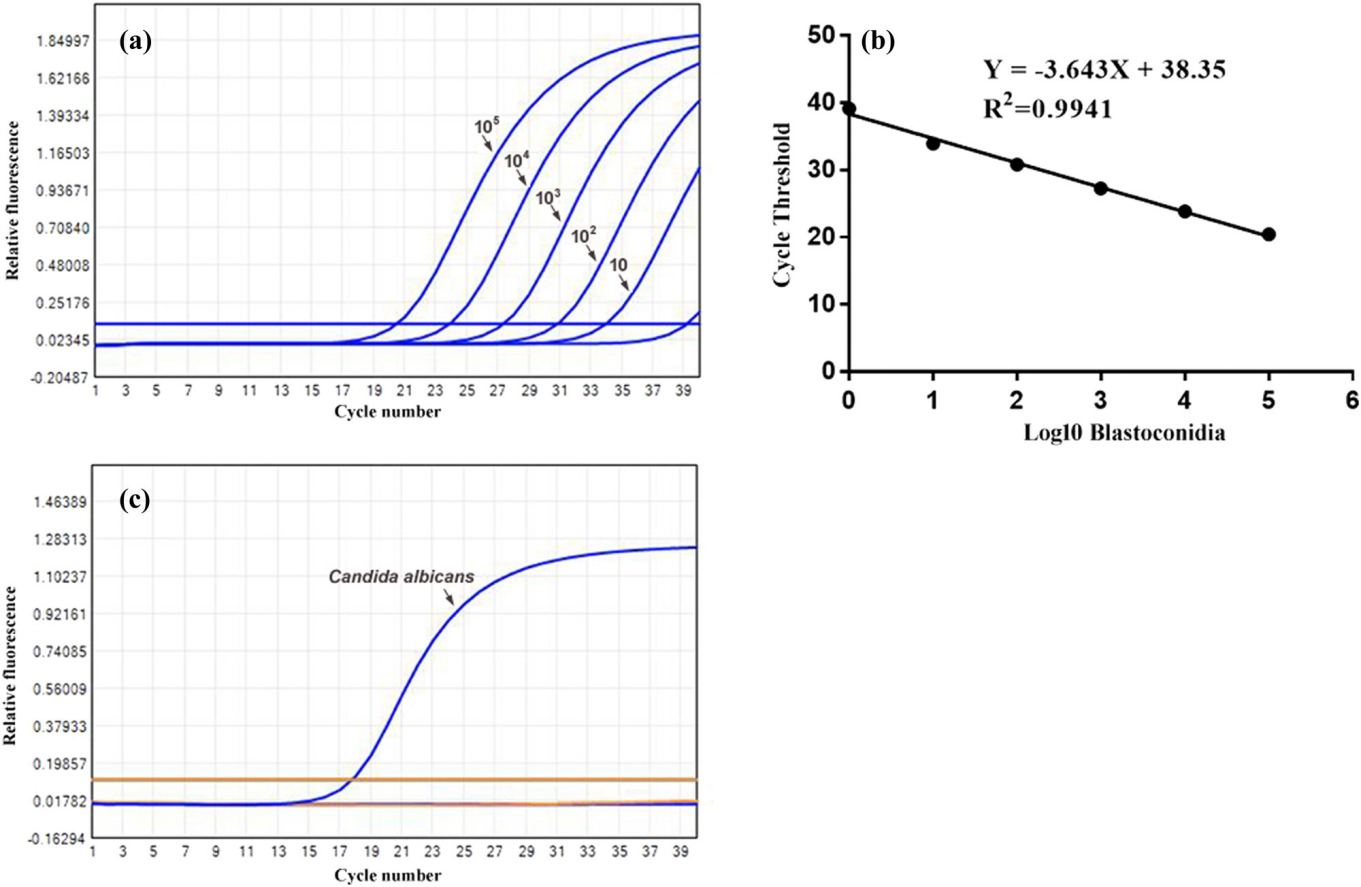
Detection sensitivity and specificity of TaqMan qPCR combined with heat-shock DNA preparation. (a) The PCR assay could detect *C. albicans* DNA as low as 10 CFU/mL. (b) The linear range of the PCR assay for the quantification of *C. albicans* was determined by the analysis of a dilution series of *C. albicans* genomic DNA. The slope of the regression line was −3.643, and the intercept was 38.35. The correlation coefficient (*R* ^2^ value) of the curve was 0.9941. (c) The PCR assay could specifically identify *C. albicans*. No signal was obtained when DNA samples prepared from non-*C. albicans* pathogens were used as negative controls.

To determine the efficiency of “heat-shock” in DNA releasing, we have used a pretreatment-free DNA extraction kit as a control. In a concentration gradient of *C. albicans*, the PCR assay could detect *Candida* DNA as low as 10 CFU/mL in samples prepared by the heat-shock protocol (Figure 1a), compared to that of 10^3^ CFU/mL in the control. The linear range of the PCR assay for the quantification of *C. albicans* was determined by analysis of a dilution series of *C. albicans* genomic DNA. The slope of the regression line was −3.643, and the intercept was 38.35. The correlation coefficient (*R* ^2^ value) of the curve was 0.9941 (Figure 1b). The cross-amplifications between primers and probe with non-*C. albicans* pathogens were also assessed to determine the specificity of the PCR assay (Figure 1c). The non-*C. albicans* pathogens included a range of *Candida* strains (e.g., *Candida tropicalis*, *Candida glabrata*, *Candida parapsilosis* and *Candida krusei*) and bacterial pathogens (e.g., *Escherichia coli*, *Staphylococcus aureus*, *Klebsiella* spp. *Enterococcus faecium* and *Staphylococcus epidermidis*). The PCR assay could correctly identify the *C. albicans* without any cross-reaction with DNA prepared from other pathogens.

### 3.2 Detection of *C. albicans* DNA in blood fractions

*C. albicans* were adjusted from 10^4^ to 10^1^ blastoconidia/mL in whole blood samples; the *C*_t_ values of DNA extracted from different blood fractions were determined after 24 h of inoculation. As indicated in Table 1, in the titration series, the signal generated from the whole blood and blood cells during the PCRs is comparable, though PCR *C*_t_ values were slightly smaller in the whole blood than in blood cells. While for plasma samples, the PCR assay could only detect signals generated by the titration of 1 × 10^4^ CFU/mL.

**Table 1:** *C*_t_ values of blood fractions at various *C. albicans* blastoconidia concentrations

Concentration of *C. albicans* (CFU/mL)	*C*_t_ values of blood fractions		
	Whole blood	Blood cells	Plasma
10^4^	25.07	27.13	37.82
10^3^	29.26	31.48	n.d.
10^2^	30.52	33.66	n.d.
10^1^	36.91	34.78	n.d.

n.d., not determined.

### 3.3 Quantification of DNA in the blood sample of systemic candidiasis mice

**Figure 2:**
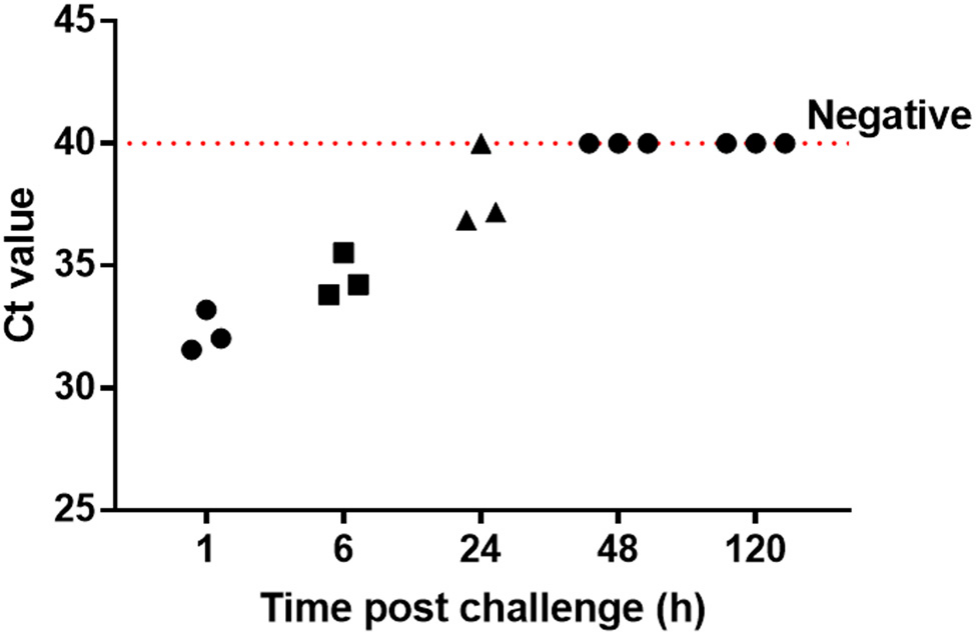
PCR test for the whole blood of IC mice. The *C*_t_ values increased gradually after *C. albicans* challenge. At the time points of 1 and 6 h, all samples were tested positive; at the time point of 24 h, two of three samples were tested positive; after 48 h, all three samples were tested negative.

Within 6 h after *C. albicans* administration, the mice showed symptoms of infection such as sweat and physical inactivity compared to the control mice. The ability of the PCR assay to measure the *C. albicans* DNA loads in the blood samples from the challenged mice was assessed. As shown in Figure 2, the *C*_t_ values increased steadily with increasing post-challenge time. All blood samples displayed strong signals by TaqMan analysis at the time point of 1 and 6 h. At the time point of 24 h, two mice were tested positive and one tested negative. None of the six samples collected at 48 and 120 h showed a positive result.

## 4 Discussion

Yeasts are a group of microbes with a vast practical potential use for humanity [17]. However, yeast-caused invasive infection (such as IC) is extremely difficult to diagnose at an early stage till today. In this study, specific primers and TaqMan probes were designed based on the ITS2 sequence of the *C. albicans* SC5314 strain. A real-time fluorescent qPCR assay was established to specifically detect *C. albicans* DNA in different samples to explore the potential for early diagnosis of IC. The limit of detection of the PCR assay was 10 CFU/mL, which was comparable to previous similar studies [18,19].

Till now, there is no standardized fungal DNA preparation protocol widely accepted by different laboratories all over the world. Unlike bacteria, viruses or mammalian cells, the solid, thick fungal cell wall which is composed of chitin, dextran, mannan and glycoproteins is hard to destroy by simple physical or chemical methods. Thus, DNA preparation has been the main obstacle for the clinical application of PCR methods in diagnosing fungal infection [20]. In addition, it was reported that 3.3% false-positive in fungal infection diagnosis was caused by DNA extraction [21]. The heat-shock DNA preparation process presented here is an easy protocol without any complex formulated reagent, which improves the *C. albicans* DNA-releasing efficiency about 100-folds compared to the samples without pretreatments. This protocol might be practical for laboratories that are not well equipped.

Which kind of specimen is suitable for real-time PCR in diagnosing IC is another controversial issue. Previous works indicated that the DNA detection performance of serum PCR is better than that of plasma and whole blood samples [22,23]. However, Loonen et al. reported that by optimizing the whole blood DNA extraction method, the PCR assay could reach a detection limit of 1 CFU/mL [24]. Our data presented here strongly suggest that the sensitivity of whole blood samples was better than that of plasma and blood cells. Although the sensitivity of blood cell sample seems to meet the detection need, the difference between the simulated specimen and the clinical infection specimen should be noted. The immune system of patients with *Candida* infection has been fighting with invading *Candida* for a long time, causing substantial *Candia* DNA released by dead *Candida* cells that exist in the plasma/serum. For the *in vitro* studies, the simulated blood sample without sufficient killing power might only lead to a small amount of DNA released out. Therefore, we recommend using whole blood samples for the detection of *Candida* spp. in designing clinical PCR protocols.

Using an established murine model [16], we have detected the *C. albicans* DNA in the blood of infected mice by TaqMan qPCR. Detectable DNA was only observed within 24 h after *C. albicans* SC5314 challenge. This means that the window period during which *Candida* DNA can be detected by PCR might be very short in IC patients. As we reported before [15], *C. albicans* could be observed in the kidney for up to 7 days, which is much longer than that in the blood presented in this study. Reasons for this may be as follows: (1) Kidneys are the major target organs following bloodstream *C. albicans* infection [25,26]. Isolated hematogenous renal infection after transient candidemia can occur, and often when renal candidiasis is suspected, blood cultures are no longer positive [27]. (2) Blood samples collected from each mouse are limited, which leads to relatively low test sensitivity.

## 5 Conclusions

Using the heat-shock DNA preparation protocol, a highly sensitive and specific PCR assay for the rapid detection of *Candida albicans* in blood was established. Based on the results presented here, we recommend using the whole blood as the optimal sample for the detection of *Candida* DNA in the blood. The window period during which *Candida* DNA can be detected by PCR might be very short in IC patients.
